# The Mucosal Immune System of Teleost Fish

**DOI:** 10.3390/biology4030525

**Published:** 2015-08-12

**Authors:** Irene Salinas

**Affiliations:** Center for Evolutionary and Theoretical Immunology (CETI), Department of Biology, MSC03 2020, University of New Mexico, Albuquerque, NM 87131, USA; E-Mail: isalinas@unm.edu; Tel.: +1-505-277-0039; Fax: +1-505-277-0304

**Keywords:** mucosal immunity, MALT, B cells, T cells, teleosts

## Abstract

Teleost fish possess an adaptive immune system associated with each of their mucosal body surfaces. Evidence obtained from mucosal vaccination and mucosal infection studies reveal that adaptive immune responses take place at the different mucosal surfaces of teleost. The main mucosa-associated lymphoid tissues (MALT) of teleosts are the gut-associated lymphoid tissue (GALT), skin-associated lymphoid tissue (SALT), the gill-associated lymphoid tissue (GIALT) and the recently discovered nasopharynx-associated lymphoid tissue (NALT). Teleost MALT includes diffuse B cells and T cells with specific phenotypes different from their systemic counterparts that have co-evolved to defend the microbe-rich mucosal environment. Both B and T cells respond to mucosal infection or vaccination. Specific antibody responses can be measured in the gills, gut and skin mucosal secretions of teleost fish following mucosal infection or vaccination. Rainbow trout studies have shown that IgT antibodies and IgT^+^ B cells are the predominant B cell subset in all MALT and respond in a compartmentalized manner to mucosal infection. Our current knowledge on adaptive immunity in teleosts is limited compared to the mammalian literature. New research tools and *in vivo* models are currently being developed in order to help reveal the great intricacy of teleost mucosal adaptive immunity and help improve mucosal vaccination protocols for use in aquaculture.

## 1. Introduction

**Figure 1:**
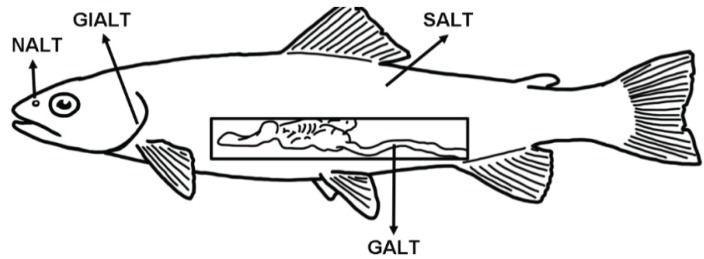
Schematic representation of the four teleost main mucosa-associated lymphoid tissues (MALT) described so far and their anatomical localization. GALT: gut-associated lymphoid tissue; SALT: skin-associated lymphoid tissue; GIALT: gill-associated lymphoid tissue; NALT: nasopharynx-associated lymphoid tissue.

Fish are continuously exposed to a microbial-rich environment (freshwater or seawater) that circulates through and reaches every epithelial barrier of their body. Thus, compared to terrestrial animals, aquatic animals have a greater challenge coping with high microbial loads, which bombard their mucosal epithelial barriers. The main mucosa-associated lymphoid tissues (MALT) of teleosts are the gut-associated lymphoid tissue (GALT), skin-associated lymphoid tissue (SALT), the gill-associated lymphoid tissue (GIALT) and the recently discovered nasopharynx-associated lymphoid tissue (NALT) (Figure 1).

When any given mucosal barrier of an animal senses a danger signal, an immediate innate immune response is triggered. This initial cue is essential for the later establishment of specific adaptive immunity. Adaptive immunity based on B and T cells and recombinatorial rearranging receptors is a canonical feature of the immune system of jawed vertebrates [1]. This double-armed B/T cell system is present in both systemic and mucosal immune systems. At the mucosal barriers, B and T lymphocytes form a dynamic network for the induction and regulation of secretory antibodies and cytotoxic T lymphocyte (CTL) responses [2]. Mucosal B cells and T cells (and their respective receptors and signaling molecules) have specialized to meet the specific demands of the mucosal environment. Generally, the mucosal immune system favors a tolerogenic microenvironment that avoids constant immune responses against non-harmful antigens present for instance in the food or microbiota. In other words, immune tolerance to maintain homeostasis is a hallmark of the mucosal environment [3].

The presence of adaptive mucosal immune responses in teleost fish has been known for decades thanks to early oral and parenteral immunization studies conducted in rainbow trout (*Oncorhyncus mykiss*) and plaice (*Pleuronectens platessa*) [4,5]. Early biochemical analyses of antibodies revealed differences in mucosal and serum immunoglobulin (Ig) molecules of fish, suggesting the presence of specialized mucosal antibodies in this group [6,7,8,9,10]. Slow progress was made for some decades until the past six years or so, when a renaissance of teleost mucosal immunity studies took place with the discovery of IgT and its function in mucosal immunity. Yet, the whole picture of how mucosal immune systems defend teleosts is far from clear. Thanks to substantial research efforts devoted to investigation of the evolution of Ig molecules in vertebrates including those present in teleosts [11,12], mucosal Igs and their function have been unraveled. However, in other realms the field of teleost mucosal adaptive immunity is clearly at its infancy. There are two major research areas that require further efforts: the biology of teleost mucosal T cells and the mechanisms by which memory is established and maintained at the mucosa.

The aim of this review is to describe the general aspects of teleost MALT anatomy as well as the adaptive immune cells, molecules and immune responses that occur at the mucosal barriers of fish including the skin, gut, gills and olfactory organ. Descriptions of the cell subsets known to be essential for mucosal adaptive immunity in mammals are included with a reference to whether or not they exist in teleosts, if known. The overview here provided should serve as a platform to encourage researchers to direct efforts towards unveiling the unique aspects of the teleost mucosal adaptive immune system. This, in turn, will lead to better mucosal vaccines for aquaculture and serve as a greater validation that teleosts are valuable models for the study of vertebrate mucosal immunity.

## 2. General Aspects of Teleost MALT Anatomy

Every vertebrate mucosal surface is armed with an associated lymphoid tissue also known as MALT. Depending on their localization in the body, MALT receive specific names. MALT appears to have first evolved as a network of diffuse leucocytes that are disseminated along the mucosal surfaces of all vertebrates. This is also known as diffuse MALT (D-MALT). On the other hand, organized lymphoid structures can be found within the mucosal epithelia of endotherms and are known as O-MALT. Some examples of O-MALT are the Peyer’s patches and tonsils. These structures are believed to have provided the anatomical, physiological and immunological basis for the maturation of antibody responses, since they provide the niche where selection for high affinity B cells clones among the entire pool of B cells takes place. O-MALT structures do not exist in teleosts (Table 1). An exception may be the curious case of the interbranchial lymphoid tissue (ILT), identified in Atlantic salmon (*Salmo salar*) (Table 1). This is a lymphocyte rich structure largely consisting of T cells embedded in a meshwork of epithelial cells, with no direct resemblance to previously described lymphoid tissues [13,14,15]. As discussed later, this structure plays a role in the immune response of salmon against viruses.

**Table 1:** Summary table of the main characteristics of teleost MALT. ? = unknown, not studied. ILT: interbranchial lymphoid tissue.

Characteristic	GALT	SALT	GIALT	NALT
**Anatomical localization**	Intestine	Skin	Gills	Olfactory organ
**Organization**	Diffuse only	Diffuse	Diffuse with one organized tissue in salmon (ILT)	Diffuse
**Presence of goblet cells**	Yes	Yes	Yes	Yes
**Total % of B cells**	4-5%	4-5%	?	35%–40%
**Approximate IgT/IgM B cell ratio**	1:1	1:1	?	1:1
**Expression of pIgR (protein level)**	Yes	Yes	?	Yes
**Compartmentalized specific IgT responses against pathogens (protein level)**	Yes	Yes	Not demonstrated	Not demonstrated
**Abundant T cells**	Yes	Yes	Yes	?
**Presence of bacterial microbiota**	Yes	Yes	Yes	Yes
**Microbiota coated by secretory immunoglobulins**	Yes	Yes	?	Yes

A total of four different MALTs have been described to date in teleosts (Figure 1 and Table 1). These are GALT, SALT, GIALT and NALT. The majority of what we know about fish MALT refers to studies from salmonids and cyprinids with an emphasis on the effects of mucosal vaccines. Teleost MALTs are composed of both innate and adaptive immune cells and molecules that work together to maintain homeostasis at the mucosa. It seems that all MALT in teleosts may operate under certain primordially conserved principles, although MALT-specific unique characteristics are likely to be unraveled and we study each of these tissues in depth. In mammals, mucosal immunologists have coined the terms “inductive mucosal site/tissue” and “effector mucosal site/tissue”. Inductive sites are those where antigens sampled from mucosal surfaces stimulate cognate naive T and B lymphocytes. Effector sites, on the other hand, are those where the effector cells after extravasation, retention, and differentiation perform their action, for instance by contributing to the formation of secretory IgA antibodies [16]. Such distinction may not be easily made in teleosts, at least based on our current body of knowledge. Due to the lack of draining lymph nodes and O-MALT, we currently believe that each MALT in teleosts may function both as an inductive and effector tissue, at least with respect to IgT specific responses. Future studies on the migration, differentiation and function of mucosal B and T cells of fish may shed new light to this question.

Stimulation of one MALT often results in responses in other distant MALT. Whereas some level of inter-connectivity exists among teleost MALT, the molecular basis for a “common mucosal immune response” at multiple sites following stimulation or vaccination at one site remains to be studied [17]. It is also worth mentioning that the Society for Mucosal Immunology (SMI) does not support the use of the term “common mucosal immune system” due to fact that it is now clear that each MALT holds some degree of compartmentalization in mammals. This is still a point of debate in teleosts and it may be true that teleost MALT are not as compartmentalized as their mammalian counterparts. However, we recommend the use of this term with caution, as suggested by the SMI.

With respect to ontogeny of adaptive immunity at mucosal barriers, it is clear that the first B and T cells appear at the mucosae much later than in primary lymphoid tissues. Additionally, studies in common carp (*Cyprinus carpio*) indicate that T cell appearance precedes that of B cells appearance in MALT [18].

## 3. Teleost Mucosal B Cells and Immunoglobulins

B cells, plasma cells and Igs have specialized to defend the complex environment that defines mucosal barriers. It appears that most vertebrates have an Ig isotype specialized in mucosal immunity [19,20]. The mucosal antibody repertoire in mammals is established by both T-dependent and T-independent mechanisms [21]. The second relies on the role of the microbiota to shape antibody production. Teleost fish have an associated microbiota in each of their mucosal barriers. How these microbial communities influence mucosal B cell biology of teleosts is largely unkown.

In the mucosal secretions of mammals, a wide diversity of different Ig isotypes is present including IgA, IgM and IgG, but IgA is the chief mucosal Ig playing a role in homeostasis, innate and adaptive immune responses [22]. Similarly, in teleost fish, both IgT and IgM are detectable at the protein level in a number of mucosal secretions using immunoblotting or ELISA (for a summary see [17]). The biology and current knowledge on teleost mucosal B cells and Igs was recently reviewed [17,23]. Measuring the ratios of IgT to IgM in plasma and in mucosal secretions was the first indicator that IgT plays a major role in mucosal immunity. Thus, in rainbow trout gut, skin and nasal mucus, the IgT/IgM ratio is much larger than in plasma in the absence of any antigenic stimulation [19,24,25].

IgT^+^ B cells are the preponderant B cell subset in GALT, SALT and NALT compared to the spleen or head kidney, where IgM^+^ B cells are the main subset [19,24,25]. The total percentage of B cells in the gut and skin of trout is ~4%–5% [19,24], whereas in the olfactory organ is ~40% [25] (Table 1). Out of this total number, in all MALT approximately half of the B cells are IgT^+^ and the other half are IgM^+^ [19,24,25], although in the skin the proportion can be up to 60%/40% [24]. This is in agreement with results from other species, for instance carp, where the percentage of B cells was estimated to be 5%–10% in the intraepithelial lymphocyte (IEL) compartment and the same in the skin and the gills [26].

The role of IgD in vertebrate mucosal immunity continues to be in many ways an enigma. The presence of a V domain associated with the trout secretory IgD molecule indicates that this isotype may potentially be involved in specific antibody responses [27]. Total IgD levels range from 2 to 80 μg/mL in the plasma of rainbow trout [27] but may be very low or below detection levels at mucosal secretions since no quantification of this Ig in mucus has been made. Total IgD secreting plasma cells were measured in systemic lymphoid tissues as well as the gills of rainbow trout and it was found that the IgD to IgM plasma cell ratio is about 1:1 in gills and approximately 4 fold lower in systemic lymphoid tissues [27]. This finding along with detection of secreted IgD transcripts in mucosally vaccinated trout may indicate a role for IgD in mucosal immunity. However, specific IgD plasma cells or specific secreted IgD in gill mucus in response to antigenic stimulation have not been measured to date. Functional experiments are critical for ascertaining the functional role of IgD in the mucosal adaptive immune response of fish.

Overall, our knowledge on plasmablasts, plasma cells and memory B cells in teleost fish MALT is very scant [17]. Previously, hydroxyurea (HU) has been used to distinguish between HU-sensitive (plasmablast) and HU-insensitive (plasma cell) activities in rainbow trout [28]. However, we currently lack specific markers that define fish memory B cell populations. Using ELISPOTs, total numbers of plasma cells from MALT have been identified in a number of fish species and mucosal tissues (reviewed in [17]). However, only antigen specific IgM secreting plasma cells have been measured. IgM and IgZ-producing cells were detected by *in situ* hybridization in the gill of mandarin fish [29]. In the same study, no IgD-producing cells were detected in the gills, adding more controversy to the potential role of IgD in gill immunity. Generally speaking, it is unclear how naïve B cells become activated and how they mature into plasmablasts and plasma cells in the mucosal tissues of fish. Moreover, the maturation of mucosal B cells into plasma cells may be governed by distinct signals in the mucosa of teleosts compared to mammals; a question that needs to be resolved in fish. It has been proposed that teleost gut has a limited number of classical plasma cells and that they are not easily detectable in the mucosal tissues [10]. Whereas long-lived plasma cells have been identified in the main lymphoid organs of teleosts, whether or not these exist in MALT is unknown.

## 4. Teleost Mucosal T Cells

Generally speaking, teleost fish have T cell populations with similar characteristics to those found in mammals. Two major T cell receptors (TCR), TCRαβ and TCRγδ have been described in teleosts. Additionally the CD4 and CD8 co-stimulatory molecules have been cloned and some antibodies against these molecules have been produced. These two molecules define the CD8^+^ and CD4^+^ T cell subsets which appear to have conserved functions in vertebrates: cytotoxic *versus* helper T lymphocytes [30]. The description of several key T cell markers including CD4, CD8, CD3, CD28, CTLA4, as well as important cytokines suggest that, similar to mammals, different T helper (Th) subtypes (Th1, Th2 and Th17) exist in teleost fish [31]. Additionally, the availability of monoclonal antibodies against the T cell markers, CD8 and CD3ε, in rainbow trout [32,33] and CD3ε in Atlantic salmon [14] has helped the study of mucosal T cells. Finally, the specific T cell monoclonal antibody DLT15 detects T cells in European seabass (*Dicentrarchus labrax*) [34] whereas the WCL38 antibody detects T cells in the common carp [26] and those two tools have been very valuable for the study of mucosal T cells in both species.

In every vertebrate, mucosal T cells possess unique features that make them particularly suitable for the mucosal microenvironment, where millions of food antigens and symbionts are present. Intestinal T cell subset development is controlled by the microbiota according to murine studies [35]. T cells appear to be very abundant in GALT, SALT and GIALT of common carp, accounting to 50%–70% of all lymphoid cells [26] and T cell markers have been found in GALT, SALT, GIALT and NALT of teleosts [10,25,26,34,36,37,38,39]. Additionally, studies in carp using WCL38 suggested that T cells from skin, gut and gills represent a distinct subset from those present in systemic lymphoid tissues [26].

Gut mucosal T cells are divided into two populations, the IEL subset and the lamina propria leucocyte (LPL) subset. In mammals, IELs are predominantly CD8^+^ T cells, whereas CD4^+^ T cells dominate in the lamina propria (LP). In teleost, IELs are also predominantly CD8^+^ T cells (reviewed by [10]). Interestingly, studies on rainbow trout showed that the gut IEL T cell repertoire is not distinct from its systemic counterpart. TCRβ transcripts of rainbow trout IELs are highly diverse and polyclonal in adult naive individuals, in sharp contrast with the restricted diversity of IEL oligoclonal repertoires described in birds and mammals [38]. Teleost IELs share similar features to systemic T cells and therefore may not represent a distinct compartment such as that present in mammals.

In GIALT, T cells may represent around 10%–20% of all lymphoid cells [33]. Additionally, the discovery of the interbranchial lymphoid tissue (ILT) in Atlantic salmon as a CD3ε rich lymphoid tissue makes this species somewhat unique among other vertebrates and supports the importance of T cells in teleost gill adaptive immunity [14,15]. In carp skin, T cell numbers are also very abundant [26] and transcription levels of T cell markers point to a generally high presence of T cells in the teleost skin. Thus far, NALT T cells have not been studied (Table 1) but it is likely that T cells are abundant in the teleost NALT.

It is clear that further studies on specific T cells and T cell functions will lead to further expansion and understanding of teleost mucosal immunity. Below, the most important T cell subsets known to play a role in the mucosal immune system of mammals are discussed. In those instances where information is available within the teleost fish literature, teleost-specific information is included.

### 4.1. Mucosal CD8 T Cells

CD8^+^ T cells comprise the majority of the IEL population of the teleost intestine, similar to what happens in mammals [10,39]. The main function of these cells in mucosal barriers is to clear pathogen-infected epithelial cells. At the transcript level, CD8α is expressed in salmonid IEL preparation [40], in gills [41] and skin [42]. The recent development of an anti-trout CD8α antibody [32] has enabled a number of in-depth studies pertaining cytotoxic T cells in this species. In the intestine and gills of rainbow trout, CD8α^+^ T cells account for 54% and 24% of all lymphocytes, respectively [32]. Interestingly, this study found numerous intra- and subepithelial CD8^+^ cells in intestine and gill (~55% and ~25%, respectively) and few scattered CD8^+^ T cells in spleen and pronephros. The high prevalence of CD8^+^ T cells in the gills is remarkable and unique to teleost respiratory organs compared to mammalian lungs, where this cell subset is present at low abundances. In trout, IELs showed potent cytotoxic activity [43]. Additionally, an anti-*Siniperca chuatsi* CD8 polyclonal antibody was published and showed presence of CD8^+^ T cells in the intestine of this species [44].

In mammals, CD8^+^ IEL are a phenotypically diverse and anatomically restricted population of lymphocytes that use γδ heterodimers for antigen recognition [45]. Similarly, rainbow trout sorted CD8α^+^ T cells express TCRγ transcripts [32]. Mucosal γδ T cells have received much attention in the mammalian literature for a number of reasons. First, γδ T cells are unique because they are the first T cells to develop in the thymus during early development. Additionally, their TCR displays very little diversity. Finally, their abundance in circulation and main lymphoid organs is low, whereas in murine IELs and murine skin can be up to 50% and 5%–40% of all cells, respectively [46,47]. These characteristics point to a key role of γδ T cells in innate mucosal immunity. Since this review focuses on adaptive immunity, we will not discuss this cell type in depth. However, it is important to mention that it has been characterized in the gut of the European seabass [48] and future studies should address the function of mucosal γδ T cells in teleost fish, particularly since they possess unexpected functional compared to those reported in mice and humans.

### 4.2. Mucosal CD4 T Cells

CD4^+^ T cells are a main component of the adaptive immune system of vertebrates. In mucosal surfaces, a number of CD4^+^ T cell subsets have been characterized. As mentioned before, the LPL population consists mostly of effector CD4^+^ T cells. In mammals, naïve CD4^+^ T cells can acquire either a Th1 or a Th2 phenotype depending on the cytokine milieu in which they become activated, however we know very little about mucosal CD4^+^ T cells in fish. In zebrafish, intestinal T cells contribute to gut homeostasis by producing cxcl8-l1 but not cxcl8-l2 [49]. In other studies, teleost skin and gills showed a high constitutive expression of Th2 markers suggesting that these two mucosal surfaces have a skewed immune response targeted against parasites [50]. Since teleosts do not produce Th2 specific Igs such as mammalian IgE, Th2 cytokines are likely to promote effective humoral responses specific against parasites. Thus far, it seems that IgT may be the isotype responsible for mucosal anti-parasite responses, although all studies conducted have used protozoan parasite models rather than helminthes or other worms known to trigger IgE-mediated immunity in mammals.

One of the key aspects of mucosal barrier homeostasis is controlling and maintaining the effector CD4^+^ T cell population at check. The latter requires the presence of key regulatory cell subsets. Thus, in mammals, regulatory T cells (Tregs) are key regulators of mucosal homeostasis [51,52]. The presence of this T cell population is modulated by cytokines released from mucosal dendritic cells and microbiota signaling. Mucosal dendritic cells produce the cytokines necessary for T cells to differentiate into Tregs. Unfortunately, we currently have no information regarding mucosal Tregs in teleosts, although the FoxP3 gene has been identified in a number of species such as carp [53] and rainbow trout [54]. Mammalian Tregs have a unique TCR repertoire that mostly recognizes the bacteria of colonic contents [55]. However, *in silico* analysis of the FoxP3 gene in trout revealed that the N-terminal required for FoxP3-mediated repression of transcription is greatly diverged between fish, amphibians and monotreme mammals compared to eutherian mammals [54]. Thus, the authors suggested that FoxP3 in fish, frog and platypus may have a different role to the human and mouse counterparts. This opens up the question: do mucosal Tregs exist in teleosts?

Mucosal tissues contain large numbers of tissue-resident memory T cells (TRM), which are believed to have a key role in barrier defense and maintenance of tissue integrity [56]. TRM are populations of clonally expanded memory T cells that permanently reside in peripheral tissues, are maintained independently of lymphoid and circulating memory T-cell populations, circulate poorly and have the ability to respond rapidly to re-exposure to cognate antigen [57]. The best-studied resident memory T cells in mammals are CD8^+^ memory T cells. However, activated CD4^+^ T cells, once they reach mucosal barrier, can also persist for long periods of time as tissue-resident memory populations. These cells clearly play key roles in regulating local immunity of mammals.

We currently have no indication that these cells exist in teleosts. Mouse CD4 TRMs are characterized by the up-regulated expression of the early activation marker CD69 and the integrin CD11a [58]. No CD69 ortholog exists in teleosts therefore alternative markers may define this population in fish. CD11a orthologs exist in zebrafish (*Danio rerio*). Thus, it is possible that with the development of antibodies to detect CD11a, teleost TRMs may be described in the future.

## 5. Adaptive Mucosal Immune Responses of Teleost Fish

Mucosal infections are very common in teleost fish and lead to both local mucosal and systemic immune responses. The clear impacts of mucosal pathogens on fish health have prompted substantial research efforts to develop mucosal vaccines for use in aquaculture. Mucosal vaccination also has other advantages over injected vaccines, particularly the ease of administration to large numbers of fish of any size. Mucosal vaccination routes tested to date in fish include immersion vaccination, oral or anal vaccination and nasal vaccination [25,59]. However, mucosal vaccines for use in aquaculture, similar to what happens in humans, are promising but present many challenges. As mentioned earlier, mucosal environments are generally tolerogenic and therefore mucosal adjuvants have been used to overcome the baseline status of the mucosal immune system to achieve high levels of protection [3].

The adaptive immune system at mucosal surfaces of mammals continues to be extensively studied. Unraveling how mucosal lymphocyte home back to mucosal tissues provided the mechanistic basis for mucosal specific compartmentalized responses. To date, homing of lymphocytes to the mucosal barriers of fish has not been demonstrated. In principle, the lack of differentiated inductive and effector mucosal sites in fish may permit a more simple mechanism of function for teleost MALT. Substantial evidence demonstrates that teleots MALT mount both B and T cell responses in response to infection or vaccination, leading to mucosal specific adaptive immunity in fish. Nevertheless, functional studies are limited to measuring specific antibodies in mucosal secretions. Specific cellular immunity in teleost MALT is yet to be shown. In any case, the question remains as to whether there is local differentiation and expansion or rather selective migration of B cells to teleost MALT. This question is very important with regards to the design of mucosal vaccines and the long-term maintenance of memory.

Undoubtedly, the route of immunization dictates the nature, length and magnitude of the host adaptive immune response. Most of our knowledge concerning teleost adaptive immunity at the mucosal level derives from immersion or oral vaccination studies where antibody titers in mucosal secretions and serum were measured. A comprehensive summary of Ig and B cell mucosal immune responses can be found in [17]. Generally speaking, it seems that all three Ig isotypes respond to a certain extent to mucosal vaccination or infection at least at the transcript level in the gut, gills, skin and olfactory organ of teleosts [25,60,61,62,63]. Measuring secreted IgM, IgT and IgD transcripts may provide some indication of the potential role of these isotypes at each mucosal surface but ultimately does not provide functional evidence for specific protection. A recent study using an attenuated *Flavobacterium psychrophilum* vaccine strain detected increased levels of secreted IgT and IgD transcripts in the gills of immersion vaccinated trout 28 days post-vaccination as well as in the gut of anally intubated fish [63]. In a different study, salmon ILT showed some delayed IgT responses at the transcript level in response to infectious salmon anaemia virus infection [64].

With regards to NALT, microarray studies revealed that IgM but not IgT transcripts as well as the polymeric Ig receptor were greatly up-regulated in the local olfactory organ following nasal vaccination. No studies have thus far shown which Ig is the main isotype responsible for nasal specific immunity (Table 1). However, as mentioned earlier, at the protein level and in the absence of any antigenic stimulus, IgT is the predominant isotype similar to what has been reported in gut, gills and skin.

At the protein level, both IgM and IgT proteins can be detected in the gut, gill, skin and nasal mucosal secretions in the absence of antigenic stimulation and following vaccination. Interestingly, all four MALT share the common feature of greater IgT to IgM ratios compared to plasma [25]. Despite the presence of specific IgM and IgT antibodies in teleost mucus, the unequivocal role for specific IgT mucosal antibodies against different gut and skin parasites has been elegantly demonstrated in rainbow trout showing compartmentalized IgT specific antibody responses in the mucosa and IgM specific antibody responses in plasma [19,24] (Table 1). As mentioned before, no inductive and effector mucosal tissues have been clearly identified in teleosts to date. In a rainbow trout skin study, it was suggested that the inductive site for the observed IgT responses is the skin because IgT-specific titers against *Ich* in infected animals can be observed only in the skin mucus and not in the serum. Further studies need to determine whether true inductive and effector mucosal sites can be delineated in teleosts.

With respect to mucosal B cell responses, in the skin of trout that survived an *Ich* infection, the numbers of IgT B cells increased ~4 fold but IgM B cell numbers remained unchanged [24]. A similar (~5 fold) increase in IgT B cell numbers was found in the gut of trout that survived a *Ceratomyxa shasta* infection [19]. Again, IgM B cell numbers were not affected in the gut of these fish. Both studies clearly support the idea that mucosal (IgT) antibody responses are specifically contained in the local environment of the mucosa whereas IgM responses have a more systemic profile.

With respect to mucosal T cells, the majority of the studies remain fairly descriptive. The majority of the studies so far published reveals the up or down regulation of T cell marker genes such as CD3, CD8 or CD4 in response to infection or vaccination. From those studies, it is clear is that mucosal T cells respond to mucosal antigenic stimulation. For instance, the numbers of CD3^+^ T cells increase in the gut of rainbow trout in response to oral vaccination with an infectious pancreatic necrosis virus vaccine [65]. However, the functional aspects of this observation remain unresolved. Significant modifications of the trout IEL TCRβ repertoire were observed after a systemic infection with a fish rhabdovirus and were especially marked for Vβ4-bearing receptors [38]. How different subsets of mucosal T cells respond to different antigens and how the microbiota contributes to mucosal T cell education is not known. Moreover, we currently have no information on how mucosal antigen presenting cells educate mucosal T cell subsets and how these T cells control the production of mucosal Igs.

In gills, both gill tissue and ILT have been studied in a number of *in vivo* models. For instance, rainbow trout CD3 expression is up-regulated in response to viral hemorrhagic septicemia infection [66]. In Japanese flounder, immersion vaccination with *Vibrio anguillarum* results in increased CD4-1, CD4-2 and CD8α expression in the gills [67]. In a recent report in Atlantic salmon challenged with infectious salmon anemia virus, the size of the ILT decreased and levels of CD3ζ transcripts increased indicating that this structure plays a role in the antiviral immune response [15]. In the case of amoebic gill disease, salmon ILT seemed to increase in size in response to infection, however, the increase in size was explained by epithelial hyperplasia rather than expansion of the T cell populations in response to infection [68].

In rainbow trout skin, CD8^+^ T cells were examined by immunohistochemistry for a period of 14 days following infection with *Ichthyobodo necator.* This study revealed the skin CD8^+^ T cells declined and that a “Th1-to-Th2” like switch took place in the skin as a result of this parasitic infection [69]. Contrary to this, *Gyrodactylus salaris* infection in Baltic salmon led to increased CD8α expression [42]. Finally, at the transcript level, a transient increase of TCRα (CD4-1) in Atlantic salmon skin was observed in response to salmon louse infection but other T cell markers were down-regulated [70].

In zebrafish, bath vaccination more efficiently elicited protective Th17-like immunity than injection vaccination in mucosal tissues. In the same study, bath vaccination of turbot elicited Th17-like responses in mucosal and systemic tissues [71].

## 6. Conclusions

The mucosal immune system of all jawed vertebrates relies on the role of B and T cells to mount specific adaptive immune responses that protect every mucosal surface. The field of teleost mucosal immunity is currently witnessing its renaissance. It seems clear that similar to mammals, B and T cells that defend mucosal barriers have evolved specific phenotypes and biological functions that make them particularly suitable to cope with the mucosal antigenic environment. We anticipate that novel tools that allow the study of specific T cell subsets will lead to the consolidation of the field. This kind of advance will be critical for the application of basic science to the field of mucosal vaccines for use in aquaculture. Finally, it will increase the use of teleost fish models for the study of human mucosal diseases.
